# Radioligand therapy of metastatic prostate cancer using ^177^Lu-PSMA-617 after radiation exposure to ^223^Ra-dichloride

**DOI:** 10.18632/oncotarget.15698

**Published:** 2017-02-25

**Authors:** Hojjat Ahmadzadehfar, Stefanie Zimbelmann, Anna Yordanova, Rolf Fimmers, Stefan Kürpig, Elisabeth Eppard, Florian C. Gaertner, Xiao Wei, Stefan Hauser, Markus Essler

**Affiliations:** ^1^ Department of Nuclear Medicine, University Hospital Bonn, Bonn, Germany; ^2^ Institute for Medical Biometry, Informatics and Epidemiology, University of Bonn, Bonn, Germany; ^3^ Department of Urology, University Hospital Bonn, Bonn, Germany

**Keywords:** Lu-PSMA-617, hematotoxicity, radium-223, prostate cancer, radioligand therapy

## Abstract

Radioligand therapy with ^177^Lu-PSMA-617 is an innovative and effective therapy for castrate-resistant metastatic prostate cancer patients. For patients with symptomatic bone metastases without visceral metastases, the guidelines recommend radionuclide therapy with ^223^Ra-dichloride as a single therapeutic agent or in combination with hormone therapy. The aim of this study was to evaluate the safety of repeated cycles of ^177^Lu-PSMA-617 after exposure to more cycles of ^223^Ra. Forty-nine patients were treated with three cycles of Lu-PSMA-617 divided into two groups subjected to a history of therapy with ^223^Ra. Group 1 included 20 patients, who had received therapy with ^223^Ra prior to Lu-PSMA-617 therapy. Group 2, which was the control group regarding hematotoxicity, comprised 29 patients without any history of a bone-targeted radionuclide therapy. No CTC 4° hematotoxicity was observed in the entire study population. There was no CTC 3° or CTC 4° leucopenia in either group. One and three patients from group 1 and 2, respectively, showed CTC 3° anemia. In group 1 there was significantly more CTC 2° anemia (50% vs. 6.9%) (p=0.008). One patient from group 1 (5%) showed a CTC 3° thrombocytopenia without any concurrent anemia, and two patients from group 2 (7%) showed a CTC 3° thrombocytopenia, one with CTC 3° anemia and one without any anemia. There were no significant differences between the two groups regarding leucopenia and thrombocytopenia. These results confirmed that performing repeated cycles of Lu-PSMA-617 after ^223^Ra seems to be safe with a very small probability of hematotoxicity.

## INTRODUCTION

Prostate-specific membrane antigen (PSMA) is an attractive target for the diagnosis and therapy of metastasized prostate cancer (mPC) [1–4]. So far, all of the published papers about radioligand therapy (RLT) with ^177^Lu-PSMA-617 (Lu-PSMA-617) have demonstrated that this therapy is safe and has a low toxicity profile [5–8]. For patients with symptomatic bone metastases without visceral metastases the international guidelines recommend radionuclide therapy with ^223^Ra-dichloride (^223^Ra, Xofigo^®^) as a single therapeutic agent or in combination with hormone therapy [9]. Although, according to our recently published results, performing Lu-PSMA-617 therapy after therapy with ^223^Ra seems safe with a low hematotoxicity profile [5, 7, 10, 11], because of the limited number of patients in these studies, as well as the low number of performed therapy cycles (max. 2 cycles), there was a need to evaluate hematotoxicity in patients after exposure to ^223^Ra who received 3 cycles of RLT compared to patients without a history of therapy with ^223^Ra.

## RESULTS

Altogether 147 cycles of RLT were performed. The mean follow-up time after the third cycle was 3.4 months (2-10 months). Patients in groups 1 and 2 were treated with a mean cumulative activity of 18 GBq and 17.8 GBq, respectively (*p* = 0.55) (Table 1).

**Table 1:** Pre-therapeutic and therapeutic parameters in both groups

Parameter	Group 1 (Hx of Ra-223)		Group 2		*p*-value
**n of patients**	20 (40.8%)		29 (59.2%)		
**Age (mean; +/-SD)**	71.2 (+/- 5.6) (range: 57–82)		71.3 (+/- 9.6) (range: 48–87)		0.94
**Gleason score**^1^	<=7	>7	<=7	>7	0.48
	5 (25%)	15 (75%)	8 (27.5%)	19 (65.5%)	
**laboratory findings (mean; range)**					
Hb (g/dl) (norm: 12.5–17,2)	11.1 (8.2–12.9)		11.2 (6–14.5)		0.73
WBC (G/l) (norm: 3.6-10,5)	5.4 (3.3–10.8)		6.6 (1.52–12.2)		0.07
Plt (G/l) (norm: 160–370)	219 (62–562)		271 ( 152–557)		0.07
PSA (ng/ml)	818 (5.6–5910)		377 (4.7–1180)		0.09
ALP (U/l) (norm: 34–117)	189 (50–682)		205 (36–997)		0.77
LDH (norm: < 248)	264 (151–550)		283 (146–908)		0.61
**red blood cell transfusion**^2^	5 patients (25%) 0–14 days prior to the RLT		3 patients (10%) 0,14 and 180 days prior to the RLT		0.24
**Therapy**	**Hx of n (%)**	**ongoing n (%)**	**Hx of n (%)**	**ongoing n (%)**	
Abiraterone	10 (50%)	4 (20%)	12 (41%)	8 (27.5%)	0.79
Enzalutamide	6 (30%)	7 (35%)	9 (31%)	9 (31%)	0.95
Bisphosphonate or RANKL^3^ inhibitor	3 (15%)	15 (75%)	3 (10%)	18 (62%)	0.31
Chemotherapy^4^	11 (55%)		17 (58%)		0.50
External radiation^5^	8 (40%)		13 (44.8%)		0.65
**Extent of bone met**	**n of patients (%)**		**n of patients (%)**		0.63
< 6 met	0		2 (7%)		
6–20 met	4 (20%)		7 (24%)		
> 20 met	11 (55%)		13 (45%)		
Diffus/SuperScan	5 (25%)		7 (24%)		
**Lymph node met**	14 (70%)		23 (79%)		0.43
**Liver met**	1 (5%)		5 (17%)		0.19
**Lung met**	1 (5%)		4 (14%)		0.32
**ECOG**	**n of patients (%)**		**n of patients (%)**		0.35
0	6 (30%)		10 (35%)		
1	7 (35%)		14 (48%)		
2	7 (35%)		5 (17%)		
**Amount of activity (GBq)**					
First cycle	6.1 (+/- 0.6)		5.9 (+/- 0.6)		0.32
Second cycle	6.0 (+/- 0.5)		6.0 (+/- 0.6)		0.64
Third cycle	5.8 (+/- 0.6)		5.8 (+/- 0.6)		0.99
Cumulative	18.0 (+/- 1.2)		17.8 (+/- 1.5)		0.55

Abbreviations: Hx: history; ns: nonsignificant; n: number; met: metastases; SD: standard deviation; Hb: hemoglobin; WBC: white blood cells; Plt: platelets; ALP: alkaline phosphatase; ECOG: Eastern Cooperative Oncology Group performance status; RLT: radioligand therapy

^1^ Gleason score of two patients in group 2 was unclear

^2^ Red blood cell transfusion prior to the first cycle

^3^ Receptor activator of nuclear factor kappa-B ligand

^4^ in the group 1, eight patients received Docetaxel and three patients Docetaxel as well as cabazitaxel as the second line chemotherapy. In the group 2, eleven patients received docetaxel and 6 patients docetaxel as well as cabazitaxel as the second line chemotherapy

^5^ the extent of radiation to the bone metastases was in all patients in this study under 25 % of skeleton.

Although there were no significant differences regarding the number of blood cell counts between the two groups prior to the first cycle of RLT, there was more CTC 1° and 2° thrombocytopenia in patients in group 1 (*p* = 0.04) with a history of therapy with ^223^Ra (Table 2).

**Table 2:** Baseline blood values according to common toxicity criteria (version 4.0) in both groups prior to RLT

	Group 1 (Hx of Ra-223)			Group 2			
	CTC 0° (%)	CTC 1°–2° (%)	CTC 3° (%)	CTC 0° (%)	CTC 1°–2° (%)	CTC 3° (%)	*p*-value
**WBC**	17 (85)	3 (15)	0 (0)	26 (89.7)	2 (6.9)	1 (3.4)	0.47
**Hb**	3 (15)	17 (85)	0 (0)	4 (13.8)	23 (79.3)	2 (6.9)	0.48
**Plt**	14 (70)	6 (30)	0 (0)	27 (93)	2 (7)	0 (0)	0.04

Abbreviations: WBC: white blood cells, Hb: hemoglobin, Plt: platelets

### Hematotoxicity in all patients

The blood parameters according to the common toxicity criteria prior to the first cycle of therapy are listed in Table 3. Relevant anemia, thrombocytopenia and leucopenia (CTC 3°) occurred during the observation period after the third cycle in 4 (8.2%), 3 (6.1%) and 0 patients, respectively. No CTC 4° hematotoxicity was observed in the entire study population. More than 60% of patients did not show any hematotoxicity. Two patients with CTC 3° thrombocytopenia showed no anemia and one had a concurrent CTC 3° anemia. Three patients with CTC 3° anemia had no thrombocytopenia (CTC 0°).

**Table 3:** Hematotoxicity according to common toxicity criteria (version 4.0) in all patients prior to and after 3 cycles of RLT

	Baseline				After 3 cycles of RLT			
	CTC 0° (%)	CTC 1° (%)	CTC 2° (%)	CTC 3° (%)	CTC 0° (%)	CTC 1° (%)	CTC 2° (%)	CTC 3° (%)
**WBC**	43 (87.8)	4 (8.2)	1 (2.0)	1 (2.0)	42 (85.7)	5 (10.2)	2 (4.1)	0 (0)
**Hb**	7 (14.3)	33 (67.3)	7 (14.3)	2 (4.1)	30 (61.2)	03 (6.1)	12 (24.5)	4 (8.2)
**Plt**	41 (83.7)	07 (14.3)	01 (2.0)	0 (0)	37 (75.5)	07 (14.3)	2 (4.1)	3 (6.1)*

There were no treatment-associated grade 4 or 5 toxicities.

Abbreviations: WBC: white blood cells, Hb: hemoglobin, Plt: platelets

* Two patients with grade 3 thrombocytopenia showed no anemia and one had grade 3 anemia

### Hematotoxicity in each group

Table 4 shows the hematotoxicity in each group according to the CTC criteria. There was no CTC 3° or CTC 4° leucopenia in either group. One and three patients from group 1 and 2, respectively, showed CTC 3° anemia. In group 1 there was significantly more CTC 2° anemia (50% *vs*. 6.9%) (*p* = 0.008). One patient from group 1 (5%) showed a CTC 3° thrombocytopenia without any concurrent anemia (CTC 0°), and two patients from group 2 (7%) showed a CTC 3° thrombocytopenia, one with CTC 3° anemia and one without any anemia (CTC 0°). There were no significant differences between the two groups regarding leucopenia and thrombocytopenia. There was no significant correlation between the number of performed 223Ra therapies and higher hematotoxicity in group 1. There was also no significant increase in hematotoxicity rate in patients with a history of external radiotherapy compared to patients without this therapy in either group (groups 1&2).

**Table 4:** Hematotoxicity in both groups after 3 cycles of RLT

	Group 1 (Hx of Ra-223)				Group 2				
	CTC 0° (%)	CTC 1° (%)	CTC 2° (%)	CTC 3° (%)	CTC 0° (%)	CTC 1° (%)	CTC 2° (%)	CTC 3° (%)	*p*-value
**WBC**	15 (75)	3 (15)	2 (10)	0 (0)	27 (93.1)	2 (6.9)	0 (0)	0 (0)	0.12
**Hb**	8 (40)	1 (05)	10 (50)	1 (05)	22 (75.8)	2 (6.9)	2 (6.9)	3 (10.4)	0.008
**Plt**	16 (93.8)	2 (10)	1 (5.0)	1 (5.0)*	21 (72.4)	5 (17.2)	1 (3.4)	2 (7.0)+	0.88

Abbreviations: WBC: white blood cells, Hb: hemoglobin, Plt: platelets

* This patient has grade 0 anemia

+ One patient with grade 3 anemia and one with grade 0

### Hematotoxicity in patients who underwent both chemotherapy and radionuclide therapy with ^223^Ra

Eleven patients from group 1 (55%) had received chemotherapy prior to or after ^223^Ra. The median time interval between the last cycle of chemotherapy and the first cycle of the PSMA therapy was 11 months (2 - 38 months). There was no significant increase in hematotoxicity rate in these patients compared to patients in group 1 who had not received any chemotherapy (Table 5).

**Table 5:** Hematotoxicity in group 1 in patients with/without history of chemotherapy

	Group 1: Ra-223 + chemotherapy				Group 1: only Ra-223				
	CTC 0° (%)	CTC 1° (%)	CTC 2° (%)	CTC 3° (%)	CTC 0° (%)	CTC 1° (%)	CTC 2° (%)	CTC 3° (%)	*p*-value
**WBC**	8 (72.7)	2 (18.2)	1 (9.1)	0	7 (77.8)	1 (11.1)	1 (11.1)	0	0.90
**Hb**	5 (45.5)	1 (9.1)	5 (45.5)	0	3 (33.3)	0	5 (55.6)	1 (11.1)	0.50
**Plt**	8 (72.7)	1 (9.1)	1 (9.1)	1 (9.1)	8 (88.9)	1 (11.1)	0	0	0.61

Abbreviations: WBC: white blood cells, Hb: haemoglobin, Plt: platelets

### The impact of bone involvement extension on hematotoxicity

Table 6 shows the grade of toxicity according to the extent of bone involvement in each group and in all patients together. There was a significant correlation between the extent of bone involvement and thrombocytopenia 1°-3° in all patients (*p* = 0.03); however, there was just a trend toward more thrombocytopenia 1°-3° in patients in group 2 with SuperScan (*p* = 0.08). Otherwise, there was no significant increase in hematotoxicity according to the extent of bone involvement in this patient cohort.

**Table 6:** Grade of toxicity according to extent of bone involvement in both groups

	Number of bone metastases	Platelets CTC grade (%)			*p*	WBC CTC grade (%)			*p*	Hb CTC grade (%)			*p*
		0	1–2	3–4*		0	1–2	3–4*		0	1–2	3–4*	
**Group 1 (Ra)**	**< 6 met**	0	0	0	0.37	0	0	0	0.95	0	0	0	0.08
	**6–20 met**	4 (100)	0	0		3 (75)	1 (25)	0		4 (100)	0	0	
	**> 20 met**	9 (81.8)	2 (18.2)	0		8 (72.7)	3 (27.3)	0		3 (27.3)	7 (63.6)	1 (9.1)	
	**SuperScan**	3 (60)	1 (20)	1 (20)		4 (80)	1 (20)	0		1 (20)	4 (80)	0	
**Group 2**	**< 6 met**	2 (100)	0	0	0.08	2 (100)	0	0	0.37	2 (100)	0	0	0.39
	**6–20 met**	6 (85.7)	1 (14.3)	0		7 (100)	0	0		5 (71.4)	2 (28.6)	0	
	**> 20 met**	11 (84.6)	2 (15.4)	0		12 (92.3)	1 (7.7)	0		11 (84.6)	0	2 (15.4)	
	**SuperScan**	2 (28.5)	3 (42.9)	2 (28.5)		6 (85.7)	1 (14.3)	0		4 (57.1)	2 (28.6)	1 (14.3)	
**All patients**	< 6 met	2 (100)	0	0	0.03	2 (100)	0	0	0.86	2 (100)	0	0	0.39
	**6–20 met**	10 (90.9)	1 (9.1)	0		10 (90.9)	1 (9.1)	0		9 (81.8)	2 (18.2)	0	
	**> 20 met**	20 (83.3)	4 (16.7)	0		20 (83.3)	4 (16.7)	0		14 (58.3)	7 (29.2)	3 (12.5)	
	**SuperScan**	5 (41.7)	4 (33.3)	3 (25)		10 (83.3)	2 (16.7)	0		5 (41.7)	6 (50.0)	1 (8.3)	

*No CTC 4°

### Treatment response

Thirty-three patients (67.3%) showed a PSA decline 2 months after the third cycle, of whom 26 (53.1%) showed a PSA decline > 50%. Twelve (60%) and 21 (72.4%) patients from group 1 and 2, respectively, showed a PSA decline (*p* = 0.27), among whom nine patients (45%) from group 1 and 17 patients (58.6%) from group 2 showed a PSA decline > 50% (*p* = 0.26).

## DISCUSSION

Currently there are different approved drugs for castrate-resistant mPC patients, all with a positive effect on overall survival (abiraterone, enzalutamide, docetaxel, cabazitaxel, and ^223^Ra) [12–17]. ^223^Ra is approved for the treatment of patients with symptomatic bone metastases without visceral metastases [18–22]. Apart from these approved therapies, the first published data showed promising results for RLT with Lu-PSMA-617 with a low toxicity profile, which has been performed normally as the last therapy option [5–7, 10, 23, 24]. The first results showed a positive effect on the prolongation of overall survival as well [23].

A combination of the mentioned therapies, especially chemotherapeutic agents and radionuclides, may be hematotoxic, i.e. inducing a bone marrow failure (BMF). BMF may be the result of anemia, thrombocytopenia, leucopenia, or a combination of 2 or more of these factors. However, depending on the extent of bone and bone marrow metastases, bone marrow function might become compromised, resulting in anemia and thrombocytopenia [25], independently of therapeutic regimen.

Apart from this, there are some other factors that may influence the bone marrow function in patients with PC, such as castration and androgen blockage, which have been shown to cause anemia and could be corrected with recombinant erythropoietin [26, 27]. Nieder et al. showed that about half of PC patients experience low hemoglobin ( < 10 g/dL) unrelated to adverse effects of the previously mentioned therapeutic agents, and this in turn leads to a short survival time [28]. In the case of repeated radionuclide therapies with one or more radiopharmaceuticals the probability of a BMF increases, and in patients with a high tumor involvement of bone marrow, this toxicity may occur earlier with more serious consequences [29–32].

In this study no patient showed a CTC 3° leucopenia and only in 3 patients (6.1%) was a CTC 3° thrombocytopenia detected, two of them (4.1%) without a concurrent anemia (CTC 0°), which was most likely induced by RLT, and one patient with concurrent CTC 3° anemia, which was possibly induced by RLT (Table 3). In the subgroup analysis, despite there being more patients with CTC 1°/2° thrombocytopenia in group 1 prior to the first cycle of RLT, which was most likely because of the therapies performed with ^223^Ra, only one patient from group 1 showed a CTC 3° thrombocytopenia without a concurrent anemia (CTC 0°), which was probably induced by RLT, and two patients in group 2 showed a CTC 3° thrombocytopenia, one with concurrent CTC 3° anemia and one without anemia. There was no significant difference between these two groups regarding radiation-induced hematotoxicity (*p* = 0.88; Table 4). These results reinforce the fact that RLT using Lu-PSMA can be performed after ^223^Ra safely.

All of these three patients with CTC 3° thrombocytopenia had a diffuse bone and bone marrow involvement (SuperScan), which shows the higher probability of hematotoxicity in this group of patients (Table 6); however, five and four other patients with a SuperScan showed CTC 0° and CTC 1°/2° thrombocytopenia, respectively, which indicates the safety of RLT in patients in such an advanced stage of the disease. Three patients (6.1%) with CTC 3° anemia had no thrombocytopenia (CTC 0°), which was most likely due to disease progression in these patients.

According to the results of ALSYMPCA trial [33] in patients not treated with docetaxel, the hematoxicity of Ra-223 was same as that in Placebo arm showing clearly that ^223^Ra alone is not bone marrow toxic. In the current study, we did not detect any higher hematotoxicity in patients with a history of ^223^Ra and chemotherapy prior to Lu-PSMA therapy. In the group 1, eight patients received Docetaxel and three patients Docetaxel as well as cabazitaxel as the second line chemotherapy. In the group 2, eleven patients received docetaxel and 6 patients docetaxel as well as cabazitaxel as the second line chemotherapy. There was no significant increase in hematotoxicity rate because of prior chemotherapy in either group.

In our recently published paper we showed that a prior therapy with ^223^Ra had no negative impact on therapy response of a RLT [34]. In the current study a significant response after 3 cycles of RLT, defined as a PSA decline > 50%, was detected in 45% and 58.6% of patients in group 1 and 2, respectively. It seems that more patients in group 2 showed a significant response to the therapy, however this difference was not significant.

Although this study is a retrospective study and should be confirmed by prospective studies with more patients, these results encourage us to perform RLT in patients with a history of ^223^Ra therapy. Performing repeated cycles of Lu-PSMA-617 after ^223^Ra seems to be safe, with a very small probability of hematotoxicity and at the same time a significant therapeutic response. In conclusion RLT with Lu-PSMA-617 after radiation exposure to ^223^Ra is a safe with no increased probability of radiation-induced hematotoxicity same as patients without a history of treatment with ^223^Ra.

## MATERIALS AND METHODS

### Patients

Forty-nine hormone refractory mPC patients with distant metastases and progressive disease according to the PSA level were treated with three cycles of Lu-PSMA-617 with 8-week intervals between the cycles. All of these patients had at least 2 months follow-up after the third cycle. The patients were divided into two groups. Group 1 included 20 patients (40.8%), who had received between 1 and 6 cycles of therapy with ^223^Ra (median: 6 cycles; mean: 4.9) 30-365 days (mean: 106 days; median: 75 days) prior to Lu-PSMA-617 therapy. Group 2, which was the control group regarding hematotoxicity, comprised 29 patients (59.2%) without any history of a bone-targeted radionuclide therapy. There were no significant differences between these two groups of patients regarding age, Gleason score, complete blood counts, the number of red blood cell transfusions prior to the first cycle of RLT, previous therapies, the extent of bone and other metastases and the ECOG status, as well as the amount of administered activity in each cycle and cumulatively (Table 1). The local ethics committee approved this retrospective study, and all subjects signed a written informed consent.

### Laboratory tests

One day prior to each cycle, the hematological status was evaluated in all patients. The baseline blood values of each group according to the common toxicity criteria are shown in Table 2. Laboratory examinations for at least up to 8 weeks after the third cycle were obtained in all patients.

### RLT

PSMA-ligand (PSMA-617) was obtained from ABX GmbH (Radeberg, Germany). The preparation of Lu-PSMA-617 was explained in detail in a previous publication [5].

The treatment solution was administered by slow intravenous injection over 1 minute followed by 1000 ml of NaCl or Ringer's solution. All patients were discharged 48 hours after therapy in accordance with the rules of the Federal Office for Radiation Protection in Germany (BfS).

### Toxicity

Toxicity was recorded using the Common Terminology Criteria for Adverse Events (CTCAE), version 4.0, and was analyzed according to the recommendation of the NCI guidelines for investigators [35]. Etchebehere et al. [32] used the following classification for classifying the reasons for bone marrow failure (BMF), which is adopted in the current study according to CTC criteria: 1) BMF is most likely due to disease progression in patients who developed CTC 3°/4° anemia with only CTC 1°/2° thrombocytopenia; 2) it is most likely induced by RLT in patients who had CTC 3°/4° thrombocytopenia with only CTC 1°/2° anemia; and 3) it is possibly induced by RLT in patients who developed CTC 3°/4° anemia associated with CTC 3°/4° thrombocytopenia.

### Tumor response evaluation

The tumor marker PSA was used as the main marker for the response evaluation. We classified the changes in PSA level as a decrease of more than 50% and any decline. Any increase in PSA was considered disease progression.

### Statistical analysis

Variables of interest were calculated using descriptive statistics. The *chi-square test* (χ2) was used to *compare different variables in both groups*. For the comparison of variables with a normal distribution, a *t*-test was used. Statistical analysis was performed using a commercially available software package (SPSS 22, IMB, Armonk, NY, USA). Values of *P* < 0.05 were considered significant.

## References

[1] Afshar-Oromieh A, Hetzheim H, Kratochwil C, Benesova M, Eder M, Neels OC, Eisenhut M, Kubler W, Holland-Letz T, Giesel FL, Mier W, Kopka K, Haberkorn U (2015). The Theranostic PSMA Ligand PSMA-617 in the Diagnosis of Prostate Cancer by PET/CT: Biodistribution in Humans, Radiation Dosimetry, and First Evaluation of Tumor Lesions. J Nucl Med.

[2] Afshar-Oromieh A, Malcher A, Eder M, Eisenhut M, Linhart HG, Hadaschik BA, Holland-Letz T, Giesel FL, Kratochwil C, Haufe S, Haberkorn U, Zechmann CM (2013). PET imaging with a [68Ga]gallium-labelled PSMA ligand for the diagnosis of prostate cancer: biodistribution in humans and first evaluation of tumour lesions. Eur J Nucl Med Mol Imaging.

[3] Rai BP, Baum RP, Patel A, Hughes R, Alonzi R, Lane T, Adshead J, Vasdev N (2016). The Role of Positron Emission Tomography With (68)Gallium (Ga)-Labeled Prostate-specific Membrane Antigen (PSMA) in the Management of Patients With Organ-confined and Locally Advanced Prostate Cancer Prior to Radical Treatment and After Radical Prostatectomy. Urology.

[4] Ahmadzadehfar H, Azgomi K, Hauser S, Wei X, Yordanova A, Gaertner F, Kurpig S, Strunk H, Essler M (2016). 68Ga-PSMA-11 PET as a gate-keeper for the treatment of metastatic prostate cancer with radium-223: proof of concept. J Nucl Med.

[5] Ahmadzadehfar H, Rahbar K, Kurpig S, Bogemann M, Claesener M, Eppard E, Gartner F, Rogenhofer S, Schafers M, Essler M (2015). Early side effects and first results of radioligand therapy with (177)Lu-DKFZ-617 PSMA of castrate-resistant metastatic prostate cancer: a two-centre study. EJNMMI Res.

[6] Baum RP, Kulkarni HR, Schuchardt C, Singh A, Wirtz M, Wiessalla S, Schottelius M, Mueller D, Klette I, Wester HJ (2016). 177Lu-Labeled Prostate-Specific Membrane Antigen Radioligand Therapy of Metastatic Castration-Resistant Prostate Cancer: Safety and Efficacy. J Nucl Med.

[7] Ahmadzadehfar H, Eppard E, Kurpig S, Fimmers R, Yordanova A, Schlenkhoff CD, Gartner F, Rogenhofer S, Essler M (2016). Therapeutic response and side effects of repeated radioligand therapy with 177Lu-PSMA-DKFZ-617 of castrate-resistant metastatic prostate cancer. Oncotarget.

[8] Rahbar K, Ahmadzadehfar H, Kratochwil C, Haberkorn U, Schafers M, Essler M, Baum RP, Kulkarni HR, Schmidt M, Drzezga A, Bartenstein P, Pfestroff A, Luster M (2017). German Multicenter Study Investigating 177Lu-PSMA-617 Radioligand Therapy in Advanced Prostate Cancer Patients. J Nucl Med.

[9] Cornford P, Bellmunt J, Bolla M, Briers E, De Santis M, Gross T, Henry AM, Joniau S, Lam TB, Mason MD, van der Poel HG, van der Kwast TH, Rouviere O (2016). EAU-ESTRO-SIOG Guidelines on Prostate Cancer. Part II: Treatment of Relapsing, Metastatic, and Castration-Resistant Prostate Cancer. Eur Urol.

[10] Rahbar K, Schmidt M, Heinzel A, Eppard E, Bode A, Yordanova A, Claesener M, Ahmadzadehfar H (2016). Response and Tolerability of a Single Dose of 177Lu-PSMA-617 in Patients with Metastatic Castration-Resistant Prostate Cancer: A Multicenter Retrospective Analysis. J Nucl Med.

[11] Schlenkhoff CD, Gaertner F, Essler M, Schmidt M, Ahmadzadehfar H (2016). Positive Influence of 177Lu PSMA-617 Therapy on Bone Marrow Depression Caused by Metastatic Prostate Cancer. Clin Nucl Med.

[12] Scher HI, Fizazi K, Saad F, Taplin ME, Sternberg CN, Miller K, de Wit R, Mulders P, Chi KN, Shore ND, Armstrong AJ, Flaig TW, Flechon A (2012). Increased survival with enzalutamide in prostate cancer after chemotherapy. N Engl J Med.

[13] Ryan CJ, Smith MR, Fizazi K, Saad F, Mulders PF, Sternberg CN, Miller K, Logothetis CJ, Shore ND, Small EJ, Carles J, Flaig TW, Taplin ME (2015). Abiraterone acetate plus prednisone versus placebo plus prednisone in chemotherapy-naive men with metastatic castration-resistant prostate cancer (COU-AA-302): final overall survival analysis of a randomised, double-blind, placebo-controlled phase 3 study. Lancet Oncol.

[14] Parker C, Nilsson S, Heinrich D, Helle SI, O’Sullivan JM, Fossa SD, Chodacki A, Wiechno P, Logue J, Seke M, Widmark A, Johannessen DC, Hoskin P (2013). Alpha emitter radium-223 and survival in metastatic prostate cancer. N Engl J Med.

[15] de Bono JS, Oudard S, Ozguroglu M, Hansen S, Machiels JP, Kocak I, Gravis G, Bodrogi I, Mackenzie MJ, Shen L, Roessner M, Gupta S, Sartor AO (2010). Prednisone plus cabazitaxel or mitoxantrone for metastatic castration-resistant prostate cancer progressing after docetaxel treatment: a randomised open-label trial. Lancet.

[16] Kantoff PW, Higano CS, Small EJ, Whitmore JB, Frohlich MW, Schellhammer PF (2012). Re: interdisciplinary critique of sipuleucel-T as immunotherapy in castration-resistant prostate cancer. J Natl Cancer Inst.

[17] Petrylak DP, Tangen CM, Hussain MH, Lara PN, Jones JA, Taplin ME, Burch PA, Berry D, Moinpour C, Kohli M, Benson MC, Small EJ, Raghavan D (2004). Docetaxel and estramustine compared with mitoxantrone and prednisone for advanced refractory prostate cancer. N Engl J Med.

[18] Hoskin P, Sartor O, O’Sullivan JM, Johannessen DC, Helle SI, Logue J, Bottomley D, Nilsson S, Vogelzang NJ, Fang F, Wahba M, Aksnes AK, Parker C (2014). Efficacy and safety of radium-223 dichloride in patients with castration-resistant prostate cancer and symptomatic bone metastases, with or without previous docetaxel use: a prespecified subgroup analysis from the randomised, double-blind, phase 3 ALSYMPCA trial. Lancet Oncol.

[19] Nilsson S, Franzen L, Parker C, Tyrrell C, Blom R, Tennvall J, Lennernas B, Petersson U, Johannessen DC, Sokal M, Pigott K, Yachnin J, Garkavij M (2007). Bone-targeted radium-223 in symptomatic, hormone-refractory prostate cancer: a randomised, multicentre, placebo-controlled phase II study. Lancet Oncol.

[20] Parker C, Sartor O (2013). Radium-223 in prostate cancer. N Engl J Med.

[21] Sartor O, Coleman R, Nilsson S, Heinrich D, Helle SI, O’Sullivan JM, Fossa SD, Chodacki A, Wiechno P, Logue J, Widmark A, Johannessen DC, Hoskin P (2014). Effect of radium-223 dichloride on symptomatic skeletal events in patients with castration-resistant prostate cancer and bone metastases: results from a phase 3, double-blind, randomised trial. Lancet Oncol.

[22] Ahmadzadehfar H, Schlenkhoff CD, Rogenhofer S, Yordanova A, Essler M (2016). 68Ga-PSMA-11 PET Represents the Tumoricidal Effect of 223Ra in a Patient With Castrate-Resistant Metastatic Prostate Cancer. Clin Nucl Med.

[23] Rahbar K, Bode A, Weckesser M, Avramovic N, Claesener M, Stegger L, Bogemann M (2016). Radioligand Therapy With 177Lu-PSMA-617 as A Novel Therapeutic Option in Patients With Metastatic Castration Resistant Prostate Cancer. Clin Nucl Med.

[24] Kratochwil C, Giesel FL, Stefanova M, Benesova M, Bronzel M, Afshar-Oromieh A, Mier W, Eder M, Kopka K, Haberkorn U (2016). PSMA-Targeted Radionuclide Therapy of Metastatic Castration-Resistant Prostate Cancer with 177Lu-Labeled PSMA-617. J Nucl Med.

[25] Khafagy R, Shackley D, Samuel J, O’Flynn K, Betts C, Clarke N (2007). Complications arising in the final year of life in men dying from advanced prostate cancer. J Palliat Med.

[26] Nalesnik JG, Mysliwiec AG, Canby-Hagino E (2004). Anemia in men with advanced prostate cancer: incidence, etiology, and treatment. Rev Urol.

[27] Strum SB, McDermed JE, Scholz MC, Johnson H, Tisman G (1997). Anaemia associated with androgen deprivation in patients with prostate cancer receiving combined hormone blockade. Br J Urol.

[28] Nieder C, Haukland E, Pawinski A, Dalhaug A (2010). Anaemia and thrombocytopenia in patients with prostate cancer and bone metastases. BMC Cancer.

[29] Tu SM, Kim J, Pagliaro LC, Vakar-Lopez F, Wong FC, Wen S, General R, Podoloff DA, Lin SH, Logothetis CJ (2005). Therapy tolerance in selected patients with androgen-independent prostate cancer following strontium-89 combined with chemotherapy. J Clin Oncol.

[30] Miederer M, Thomas C, Beck J, Hampel C, Krieger C, Baque PE, Helisch A, Schreckenberger M (2015). Haematopoietic toxicity of radium-223 in patients with high skeletal tumour burden. Nuklearmedizin.

[31] Etchebehere EC, Araujo JC, Fox PS, Swanston NM, Macapinlac HA, Rohren EM (2015). Prognostic Factors in Patients Treated with 223Ra: The Role of Skeletal Tumor Burden on Baseline 18F-Fluoride PET/CT in Predicting Overall Survival. J Nucl Med.

[32] Etchebehere EC, Araujo JC, Milton DR, Erwin WD, Wendt RE, Swanston NM, Fox P, Macapinlac HA, Rohren EM (2016). Skeletal Tumor Burden on Baseline 18F-Fluoride PET/CT Predicts Bone Marrow Failure After 223Ra Therapy. Clin Nucl Med.

[33] Vogelzang NJ, Coleman RE, Michalski JM, Nilsson S, O’Sullivan JM, Parker C, Widmark A, Thuresson M, Xu L, Germino J, Sartor O (2017). Hematologic Safety of Radium-223 Dichloride: Baseline Prognostic Factors Associated With Myelosuppression in the ALSYMPCA Trial. Clin Genitourin Cancer.

[34] Ferdinandus J, Eppard E, Gaertner FC, Kurpig S, Fimmers R, Yordanova A, Hauser S, Feldmann G, Essler M, Ahmadzadehfar H (2017). Predictors of Response to Radioligand Therapy of Metastatic Castrate-Resistant Prostate Cancer with 177Lu-PSMA-617. J Nucl Med.

[35] National Cancer Institute Guidelines For Investigators (2013). Adverse event reporting requirements for DCTC DCTD (CTEP and CIP) and DCP INDs and IDEs.

